# Analysis of Biomarker Levels in Nasopharyngeal Swabs, Serum, and Saliva Across Different Health Conditions

**DOI:** 10.3390/life15020324

**Published:** 2025-02-19

**Authors:** Mina Pencheva, Neshka Manchorova-Veleva, David Baruh, Georgi Rusinov, Lyubomir Vangelov

**Affiliations:** 1 Department of Medical Physics and Biophysics, Faculty of Pharmacy, Medical University of Plovdiv, 4002 Plovdiv, Bulgaria; 2 Department of Operative Dentistry and Endodontics, Faculty of Dental Medicine, Medical University of Plovdiv, 4002 Plovdiv, Bulgaria; neshka.manchorova@mu-plovdiv.bg (N.M.-V.); lyubomir.vangelov@mu-polvdiv.bg (L.V.); 3 Department of Software Engineering, Faculty of Mathematics and Informatics, Sofia University “St. Kliment Ohridski”, 1164 Sofia, Bulgaria; dbaruh@uni-sofia.bg; 4 Clinic of Infectious Diseases, University Hospital St. George JSC in Plovdiv, 4021 Plovdiv, Bulgaria; georgirus@icloud.com

**Keywords:** ACE2, biomarkers, body fluid, COVID-19, infectious diseases

## Abstract

Background: Coronavirus disease 2019 (COVID-19) is associated with a wide variety of clinical manifestations. Aim: This study aims to evaluate the levels of angiotensin-converting enzyme 2 (ACE2), metalloprotease 17 (ADAM17), Interleukin-17A (IL-17A), transmembrane serine protease 2 (TMPRSS2), apelin (AP), and vitamin D (VD) biomarkers in nasopharyngeal swab (NPS), serum, and saliva, as well as the change in their values depending on the health status of individuals. Material and methods: The analysis was performed by using enzyme-linked immunosorbent assay (ELISA) methods. Results: Comparing the levels of the investigated markers in saliva, we found significantly elevated ACE2 values in vaccinated patients, followed by those with severe COVID-19, compared to healthy, previously infected, and mild COVID-19 groups. For TMPRSS2, IL-17A, ADAM-17, and AP, values were significantly higher in all non-healthy groups (previously infected, mild, and severe COVID-19) compared to healthy individuals. Serum levels of VD were consistently low across all five studied groups, suggesting values below normal ranges. Analysis of marker data in saliva, NPS, and serum revealed a positive correlation between NPS and serum and saliva and serum, as well as between saliva and NPS for all studied markers. Conclusions: In summary, monitoring changes in biomarkers present in Saliva holds promise as a predictive tool for various diseases. This approach enables the early implementation of preventive measures and protective strategies, potentially improving overall health outcomes.

## 1. Introduction

Severe acute respiratory syndrome virus (SARS-CoV-2) was initially identified in the Wuhan region of China [1]. The rapid global spread of the virus and its significant impact on public health necessitated unprecedented measures. However, due to its airborne transmission, traditional containment efforts proved challenging [1,2].

At the onset of the pandemic, there were varying opinions regarding the most effective method for detecting SARS-CoV-2, with saliva and NPS being considered [3].

Saliva, serum, and nasopharyngeal swabs (NPS) are essential tools for detecting and monitoring SARS-CoV-2 infection, each offering distinct advantages [4,5]. NPS remains the gold standard for COVID-19 testing due to its high accuracy when processed via RT-PCR, despite limitations like false negatives exceeding 30% due to improper sampling or low viral load [6]. In contrast, saliva has emerged as a promising alternative due to its non-invasive, self-collectable nature, making it ideal for rapid, large-scale testing [7,8]. Rich in biomarkers, saliva not only aids in early disease detection but also carries the SARS-CoV-2 virus, as oral cells containing ACE2 receptors act as viral entry points [9].

Despite advances in diagnostic methods [6], the sensitivity of rRT-PCR in saliva is not yet widely accepted. ELISA has emerged as a more sensitive method for detecting viral biomarkers such as proteins and peptides. Ongoing studies are investigating the comparative analysis of SARS-CoV-2 proteins in saliva using ELISA, with similar research conducted for other viral infections like Zika [7]. The growing utility of saliva in diagnostic testing highlights the importance of developing point-of-care (POC) tests, rapid tests, and standardized studies in clinical laboratories [5,8].

Combining saliva and NPS with serum biomarkers provides a comprehensive view of viral dynamics and immune response. The mucosal epithelium of the oral cavity serves as a crucial barrier, primarily consisting of multilayered nonkeratinized squamous epithelium and an underlying lamina propria [10]. Immunohistochemical studies have shown the presence of ACE2, TMPRSS2, IL-17A, ADAM-17, VD, and AP in the epithelial layer of the oral mucosa. These components are differentially expressed across regions of the oral cavity, including the buccal mucosa, lips, palate, tongue, gums, taste receptors, and serous acini [11,12,13,14,15].

ACE2, a protein expressed by nearly all cell types, plays a pivotal role in regulating the renin–angiotensin system (RAS) [16]. The binding of SARS-CoV-2’s spike protein to ACE2 leads to dysregulation, contributing to acute respiratory distress syndrome (ARDS), hypertension, and the overproduction of cytokines such as IL-1β, IL-17A, and IL-18 [17]. ADAM-17, another key regulator in cellular processes, has been shown to increase in the upper respiratory tract, where it modulates the release of soluble ACE2 (sACE2) and stimulates Th17 immune responses, further contributing to inflammation in COVID-19 [18]. Several reports have associated the increased IL-17A levels and Th17 response in the upper and lower respiratory tracts of COVID-19 patients with COVID-19 severity. The spike protein is also activated by host proteases, including TMPRSS2 and Furin, facilitating viral entry [17,18].

AP, a substrate for ACE2, has been linked to protective effects in cardiovascular and cerebrovascular diseases, which are common complications of COVID-19 [19]. Additionally, VD, synthesized in the epidermis and activated in the liver and kidneys, plays a critical role in immune modulation by binding to the VD receptor (VDR). It regulates around 3% of the human genome, emphasizing its significance in immune response, growth, and metabolism [20]. As such, there is considerable interest in evaluating the role of vitamin D in the immune response to SARS-CoV-2 infection [21].

One of our hypotheses is that the levels of ACE2, TMPRSS2, IL-17A, ADAM-17, VD, and AP in saliva and NPS could correlate with their plasma levels and thus serve as non-invasive biomarkers for assessing disease severity. This study aims to expand current knowledge by comparing the expression of these key molecules across NPS, serum, and saliva samples from various patient groups, including those with moderate and severe COVID-19, recovered individuals, vaccinated persons, and healthy controls.

## 2. Materials and Methods

### 2.1. Study Design

This prospective study included a total of 223 participants, divided into five groups: patients with mild COVID-19 (n = 102), median age: 70 years (IQR: 33–81); hospitalized COVID-19 patients with severe disease (n = 66), median age: 78 years (IQR: 68–87); vaccinees (n = 20), median age: 45 years (IQR: 27–60); recovered individuals (n = 19), median age: 55 years (IQR: 29–81); and healthy individuals (n = 16), median age: 54 years (IQR: 35–69) with no clinical or immunological signs of COVID-19.

The criteria for moderate COVID-19 included symptoms such as fever, cough, and mild respiratory distress, without the need for hospitalization or supplemental oxygen. Severe COVID-19 was characterized by respiratory failure, oxygen saturation below 94%, or the need for mechanical ventilation or intensive care support [22]. Treatment of patients with COVID-19 was carried out according to a protocol approved by the Ministry of Health.

Laboratory results and data on experimental variables for all individuals are available in Supplementary Table S1.

We studied serum levels of ACE2, IL-17A, TMPRSS2, ADAM-17, AP and VD in COVID-19 patients, vaccinated individuals, healthy individuals, and recoveries. Samples were collected on day 14 after PCR confirmation or the second dose of mRNA vaccine, as well as from control groups—healthy and recovered from COVID-19 (30 days after negative test). Healthy participants had no chronic diseases. After venipuncture, serum was separated within 4 h and stored at −80 °C.

### 2.2. Sample Collection

Patients were recruited during hospitalization at the Clinic of Infectious Diseases, University Hospital St. George JSC in Plovdiv. Vaccinated, recovered, and healthy individuals were selected from the Medical University of Plovdiv, Bulgaria, between October 2021 and April 2022.

Hospitalized COVID-19 patients were confirmed to have SARS-CoV-2 infection using real-time RT-PCR (GeneLEAD VIII apparatus and COVID-19 Real Time Multiplex RT-PCR Kit, Labsystems Diagnostics Oy, Helsinki, Finland).

Vaccinated individuals received two doses of either the Pfizer-BNT162b2 (Pfizer Inc., New York, NY, USA) or the mRNA-1273 Moderna vaccine (Moderna Inc., Cambridge, MA, USA). The control group tested negative for COVID-19 using an antigen test (Aurora Biomed Inc., Vancouver, BC, Canada) and exhibited no clinical signs of active infection.

All study participants were randomly selected and were heterogeneous in terms of age, gender, and clinical presentation.

### 2.3. NPS Collection

NPS samples were collected within three days of hospitalization using a flexible mini-tip swab. The swab was inserted through the nostril until reaching the posterior nasopharyngeal wall, held for a few seconds to absorb secretions, and then slowly removed while rotating. The specimen was placed in a sterile viral transport medium (3 mL) (BKMAM Biotechnology Co. Ltd. Changde, Hunan, China) and securely sealed. All samples were immediately transported on ice to the Virological Laboratory at University Hospital St. George JSC in Plovdiv and tested within 24–48 h of collection.

### 2.4. Saliva Collection

Participants were instructed not to eat, drink, smoke, or chew gum for 30 min prior to saliva collection. Saliva was collected using the passive drool method, where participants allowed saliva to accumulate in their oral cavity and then deposited it into a sterile container. Approximately 5 mL of saliva was collected from each participant. All saliva samples underwent a freeze–thaw cycle. Initially, unprocessed saliva was aliquoted into 1.5 mL Eppendorf tubes (Sigma-Aldrich, Merck KGaA, Darmstadt, Germany) and stored at −80 °C. After thawing, saliva samples were centrifuged at 10,000× *g* for 10 min at 4 °C to remove cellular debris. The resulting supernatant was transferred to a fresh Eppendorf tube and refrozen at −80 °C until analysis.

### 2.5. Sample Processing and Storage

Blood and NPS samples were centrifuged (Shimadzu UV160A, S. No: 28006648, Kyoto, Japan) at 3000 rpm for 10 min, after which sera were collected and stored at −80 °C. Prior to analysis, the thawed homogenates were centrifuged at +4 °C for 5 min at 3000 rpm (Sigma 3K30, S. No: 76262, Sigma Laborzentrifugen GmbH, Osterode am Harz, Germany).

### 2.6. Inclusion/ Exclusion Criteria

The main inclusion criteria for this study were as follows: (a) age above 18 years; (b) ability to provide informed consent; and (c) individuals admitted and registered with COVID-19 or eligibility for vaccination according to the national COVID-19 vaccination program.

All individuals in the control group were in good health, with no symptoms of upper or lower respiratory tract infections, no history of COVID-19, and not vaccinated against COVID-19. Healthy individuals signed an informed consent and were aged above 18 years.

The exclusion criteria were (a) systemic autoimmune disorders under immunosuppressive therapy; (b) active malignancies; (c) active acute infections; (d) or other immune mediated disorders; and (e) less than 18 years old.

### 2.7. Methods

The concentrations of ACE2, IL-17A, TMPRSS2, ADAM-17, AP, and VD in the serum, NPSs, and saliva were measured using commercially available kits (Table 1).

### 2.8. Statistical Analysis

Quantitative data were analyzed using the GraphPad Prism software (GraphPad Software 8.0.1 version, Inc., La Jolla, CA, USA). The findings were presented with means, standard deviations, and 95% confidence intervals for the means. Depending on whether equal variances were assumed or not, the average values for the ACE2, ADAM17, IL-17A, TMPRSS2, AP, and VD levels in the four used groups of persons and in the three biological contexts were compared using the Bonferroni or Games–Howell Test—One-Way ANOVA Post Hoc Tests. IBM SPSS Statistics (v.25) was used for statistical analysis.

## 3. Results

We reported a change in the biomarkers profile in the group of COVID-19 patients, recovered, and vaccinated subjects (with two doses of the mRNA vaccine) in comparison to the healthy subjects.

### 3.1. Detection of ACE2, TMPRSS2, IL-17A, VD, ADAM-17, AP, and VD Levels in Human Serum

ELISA was used to evaluate the levels of the studied biomarkers. Data analysis revealed significant differences in the levels of the studied biomarkers among the different patient groups. ACE2 levels were highest in vaccinated individuals, followed by patients with severe COVID-19. Recovered patients and healthy individuals showed similar levels, while the lowest values were observed in individuals with mild COVID-19.

TMPRSS2 showed a significant decrease in levels in patients with mild COVID-19 compared to healthy individuals, while patients with severe COVID-19 showed intermediate values. The vaccinated and recovered groups had similar levels of this biomarker. Regarding IL-17A, no significant differences were found between the groups, except for recovered patients, in whom the levels were lowest. The highest values were reported in healthy individuals. VD analysis showed that recovered patients had significantly higher levels than the other groups, while the lowest levels were observed in the vaccinated. For ADAM-17, healthy individuals showed the highest levels, while patients with severe COVID-19 had the lowest values. The vaccinated and recovered groups showed similar results. For apelin, a clear difference was observed between healthy individuals, who had the highest levels, and patients with mild and severe COVID-19, who showed significantly lower values (Table 2).

### 3.2. Detection of the Studied Biomarkers by ELISA in NPS, Serum, and Saliva Samples

ELISA analysis revealed significant differences in biomarker levels depending on the type of biological sample. ACE2 showed the highest values in serum and saliva, while levels in NPS were significantly lower. The differences between groups were statistically significant. For TMPRSS2, higher levels were again observed in serum, with concentrations in saliva and NPS being significantly lower. However, the results between saliva and NPS remained relatively close. IL-17A levels followed a decreasing trend from serum to NPS and saliva, with the lowest concentrations observed in saliva. The differences between sample types were statistically significant. For VD, serum again contained the highest concentrations, while the levels in NPS were significantly lower. However, values in saliva were intermediate between those in serum and NPS. For ADAM-17, the highest values were reported in serum, while NPS and saliva showed similar but significantly lower concentrations. A similar trend was observed for apelin, where serum contained the highest levels, while values in NPS and saliva were significantly lower (Table 3).

### 3.3. Correlation Analysis Between Body Fluid Levels of Key Biomarkers, Induced by COVID-19

**Figure 1:**
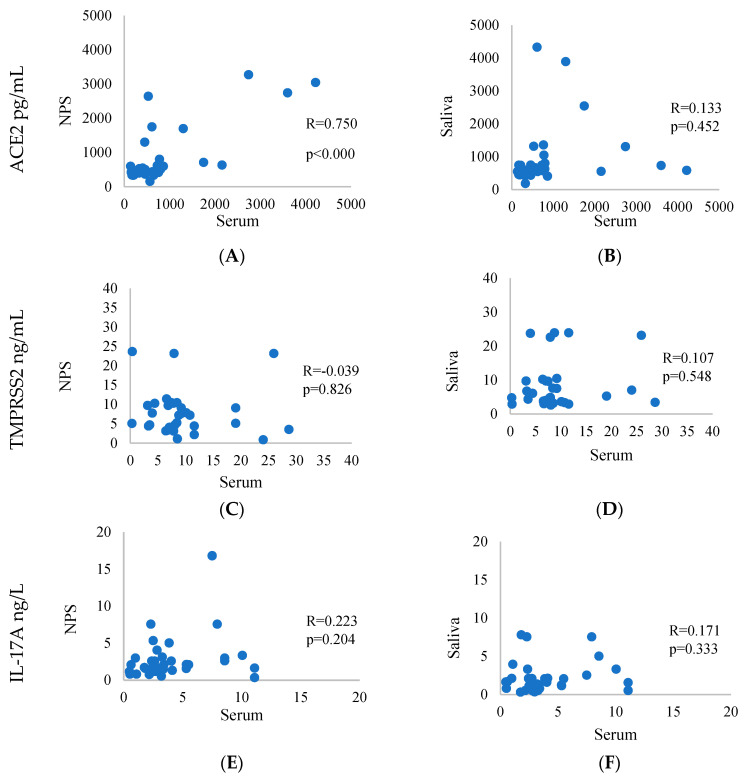

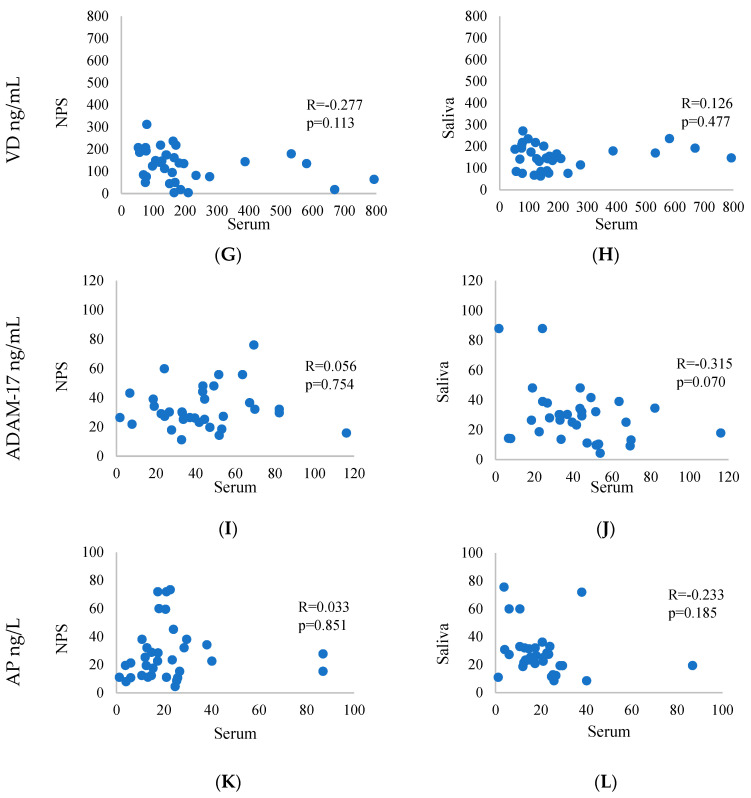
Correlation between biomarker concentrations in serum, NPS and saliva. Statistically significant values of Spearman’s rho correlation coefficient between different body fluid biomarkers. (**A**) Relation between ACE2 concentrations in NPS and serum. (**B**) Relation between ACE2 concentrations in saliva and serum. (**C**) Relation between TMPRSS2 concentrations in NPS and serum. (**D**) Relation between TMPRSS2 concentrations in saliva and serum. (**E**) Relation between IL-17A concentrations in NPS and serum. (**F**) Relation between IL-17A concentrations in saliva and serum. (**G**) Relation between VD concentrations in NPS and serum. (**H**) Relation between VD concentrations in saliva and serum. (**I**) Relation between ADAM-17 concentrations in NPS and serum. (**J**) Relation between ADAM-17 concentrations in saliva and serum. (**K**) Relation between AP concentrations in NPS and serum. (**L**) Relation between AP concentrations in saliva and serum.

The correlation analysis determined as statistically significant the measured levels of ACE2, both in total serum levels and for NPS (r = 0.750, *p* = 0.000), while between saliva and serum its influence was not well expressed (r = 0.133, *p* = 0.452). TMPRSS2 data showed a positive correlation both between serum and NPS (r = 0.039, *p* = 0.826) and between saliva and serum (r = 0.107, *p* = 0.548). For IL-1β, we found a weak correlation between NPS and serum levels, with a correlation coefficient of (r = 0.223, *p* = 0.204), indicating no statistically significant relationship. Similarly, the correlation between saliva and serum IL-1β levels was also weak, with a correlation coefficient of (r = 0.171, *p* = 0.333) (Figure 1).

About the levels for VD, we measured a negative correlation between NPS and serum (r = −0.277, *p* = 0.133) and saliva and serum (r = 0.126, *p* = 0.477). For ADAM-17, we found a rather positive correlation when comparing NPS and serum (r = 0.056, *p* = 0.754), and a weaker one between saliva and serum (r = 0.315, *p* = 0.070). The correlation of apelin was also positive in the correlation analysis between NPS and serum (r = 0.033, *p* = 0.851), and weaker between saliva and serum (r = 0.233, *p* = 0.185) (Figure 1).

## 4. Discussion

Due to the presence of SARS-CoV-2 in various bodily fluids (including nasal and pharyngeal secretions, sputum, tears, and blood), transmission occurs via saliva droplets and contaminated surfaces [23]. Changes in saliva and NPS composition may signal the early onset of COVID-19, even before typical symptoms like fever, dry cough, fatigue, and shortness of breath appear. Additionally, saliva has potential utility in predicting and diagnosing long COVID [24].

In this study, we monitored the levels of ACE2, TMPRSS2, IL-17A, ADAM-17, VD, and AP in NPS, serum, and saliva to assess their impact on health status, further supporting the need for multi-biomarker approaches in evaluating COVID-19’s effects on the immune system. The ELISA tests showed varying sensitivity depending on the biological sample, with the highest sensitivity observed in serum. In saliva, ACE2 levels were higher, while IL-17A was lowest compared to NPSs, and other markers showed similar values between NPSs and saliva. These variations align with their biological roles—ACE2 facilitates viral entry, while its shedding via TMPRSS2 and ADAM-17 generates soluble ACE2 [13,14,15,25], which may act as a decoy receptor or contribute to disease severity [26]. TMPRSS2 activation further enhances viral entry and increases Ang II levels, exacerbating RAS dysregulation and contributing to cytokine storms and microthrombosis [26,27,28,29].

Our analysis revealed significant differences in IL-17A and ADAM-17 levels (*p* < 0.05) between serum and saliva. However, no significant variations in these markers were observed among vaccinated, recovered, and COVID-19 patients. ADAM-17, on the other hand, showed significant differences (*p* < 0.05, *p* < 0.01) between healthy individuals and the other groups, suggesting its potential as a distinguishing marker for COVID-19 impact. The roles of these biomarkers are complex, as they serve protective functions under normal conditions. Our findings confirm previous studies showing higher expressions of TMPRSS2, ADAM-17, and IL-17A in healthy individuals compared to COVID-19 patients, recovered, and vaccinated individuals [30].

It is known that ADAM-17 plays a key role in cellular regulation, modulating cytokines, growth factors, and adhesion molecules [31,32,33]. In the context of COVID-19, the downregulation of ADAM-17 in infected patients could contribute to impaired immune responses, inflammation, and tissue remodeling.

The protective role of ADAM-17 in the body has been supported by other researchers. Guan et al. (2021) suggest that maintaining ADAM-17 within physiological ranges may have protective effects against cardiac fibrosis [34].

Similarly, TMPRSS2 facilitates viral entry and is upregulated by androgenic hormones, potentially explaining the higher severity of COVID-19 in men [17,34,35].

In our previous study, as well as in the research conducted by Cioccarelli et al. (2021), it was observed that the increased transcription of TMPRSS2 can be induced by elevated levels of IL-1β and TNF-α [36,37,38].

On the other hand, IL-17A is known to maintain mucosal integrity and contributes to immune responses [37,39,40,41].

The elevated levels of IL-17A observed in our study among healthy and vaccinated individuals, compared to those with severe COVID-19 and recovered individuals, likely stem from its protective function against extracellular pathogens [41] and its central role in innate immunity [40,42].

Our findings align with previous studies indicating that inadequate IL-17A levels may impair immune responses and worsen COVID-19 outcomes [43,44,45,46].

The virus-induced cytokine storm amplifies the destructive effects of TMPRSS2, ADAM-17, and IL-17A, contributing to disease severity [13,18,20,43].

At the core of COVID-19 pathogenesis lies the dysregulation of signaling and regulation of key proteins including ACE2, IL-17A, TMPRSS2, and ADAM-17, alongside reduced levels of AP and VD, subsequently impacting the progression of inflammatory processes associated with autoimmunity and inflammation modulation [35,36,44].

RAS dysregulation plays a key role in cytokine storms, neutrophil activation, and thrombotic complications [18,19,20,47]. Recent studies have suggested that AP may act as a modulator within the RAS, regulating the expression and activity of ACE2 [15,48,49,50].

Our measurements revealed elevated serum levels of AP in healthy individuals, with a positive correlation observed among healthy and vaccinated individuals, as well as those who have recovered. Previous research has indicated that hypertensive and obese patients often exhibit lower-than-normal levels of AP [51], making them more susceptible to developing COVID-19, which may progress to ARDS upon SARS-CoV-2 infection [52].

Patients with multiple comorbidities are at a heightened risk of experiencing a severe course of COVID-19 due to various factors. These individuals often exhibit increased expression of ACE2 in affected organs, likely stemming from their underlying diseases and medication regimens. Additionally, their immune systems are often compromised, further exacerbating susceptibility. Among the most prevalent comorbidities are obesity [52], diabetes [53], high blood pressure [51], heart conditions [51], etc.

Unhealthy lifestyle choices, such as smoking, further compound the risk of severe COVID-19 [54,55,56].

AP, secreted from various tissues, exhibits cardioprotective and anti-inflammatory effects. Animal studies suggest that AP/APJ signaling improves lung function and mitigates inflammatory damage, making it a potential therapeutic target for COVID-19 complications [57,58,59,60,61].

While Table 2 shows that VD levels are slightly lower in healthy subjects compared to patients with severe COVID-19, it is important to note that VD deficiency is associated with worse outcomes in COVID-19. This discrepancy may suggest that low levels of VD may impair immune function, potentially leading to severe disease. There is a possibility that, although VD levels are higher in severe patients with COVID-19, this may be due to treatment effects [62,63]. Additionally, the smaller number of healthy study participants may also explain this difference.

Across all groups studied, VD levels were consistently low. The only significant difference was observed between vaccinated individuals and those who had recovered. These consistently low VD levels suggest a widespread deficiency in the population. VD deficiency is common throughout Europe, with rates ranging from 50% to 70% and up to 90% in some countries like Poland [64,65]. VD deficiency can impair its immunomodulatory function, leading to immune system dysregulation, as seen in COVID-19 [16]. It is considered one of the factors contributing to COVID-19 severity and complications [66,67]. Our study possesses several limitations. Firstly, it encompasses a relatively small cohort of patients, underscoring the necessity for validation in larger-scale studies. Secondly, our access to samples from severe COVID-19 cases was limited solely to patients in the intensive care unit.

Furthermore, the number of vaccinated and healthy individuals in our study was small due to prevailing vaccine hesitancy during the study period. Additionally, gender segregation further diminished the participant pool within each group.

## 5. Conclusions

Our study highlights the importance of saliva and NPS samples as early indicators of COVID-19. Key biomarkers such as ACE2, TMPRSS2, IL-17A, ADAM-17, and VD were evaluated across groups, showing their critical roles in immune regulation and disease severity. Notably, low VD levels were prevalent in all groups, indicating a widespread deficiency with potential implications for COVID-19 outcomes. Additionally, elevated levels of AP in healthy individuals suggest its protective role and therapeutic potential.

The analysis shows that the biomarkers studied have similar values in saliva and NPSs. However, saliva stands out as a more reliable and convenient sampling method, it provides high sensitivity, can be administered independently and does not require specialized personnel.

Future studies should aim to validate these findings in larger cohorts and investigate the clinical applicability of these biomarkers in non-invasive sample collection settings.

## Figures and Tables

**Table 1:** Sensitivity and assay range of biomarkers measured with ELISA Kit.

ELISA Kit	Sensitivity	Assay Range
ACE2 (pg/mL)	9.825	62.5–4000
ADAM-17 (ng/mL)	0.67	0.75–200
TMPRSS2 (ng/mL)	0.137	0.15–32
Vitamin D (ng/mL)	2.114	2.5–600
Apelin (ng/L)	0.756	1–200
IL-17A (ng/L)	0.05	0.1–20

**Table 2:** Assessment of biomarker levels by ELISA.

Biomarker	Group	Means	IQR, min/max	*p*-Value
ACE2 (pg/mL)	Healthy	320	157.7–483.6	0.008
	Recovered	348	179.4–516.5	0.004
	Mild COVID-19	137.5	117.8–157.2	<0.001
	Severe COVID-19	1757	1311–2203	<0.001
	Vaccinated	2190	1718–2662	<0.001
TMPRSS2 (ng/mL)	Healthy	14.98	11.28–18.67	<0.001
	Mild COVID-19	5.77	4.84–6.70	<0.001
	Severe COVID-19	12.53	9.84–15.21	<0.01
	Vaccinated	10.46	7.55–13.36	<0.01
	Recovered	9.91	7.55–12.28	<0.01
IL-17A (ng/L)	Healthy	4.16	2.06–6.27	<0.001
	Recovered	2.47	1.23–3.71	<0.05
	Mild COVID-19	3.79	2.91–4.66	<0.05
	Severe COVID-19	3.19	2.26–4.12	<0.01
	Vaccinated	4.05	2.89–5.22	0.15
Vitamin D (ng/mL)	Healthy	143.9	128.0–159.8	0.09
	Recovered	234.9	190.2–279.5	<0.05
	Mild COVID-19	211.6	141.5–281.6	<0.05
	Severe COVID-19	156.6	117.4–195.8	<0.05
	Vaccinated	123.2	105.4–141.0	0.110
ADAM-17 (ng/mL)	Healthy	129.1	82.07–176.0	<0.001
	Recovered	53.03	23.64–82.42	<0.01
	Mild COVID-19	43.41	35.39–51.24	<0.05
	Severe COVID-19	36.42	30.08–42.76	<0.01
	Vaccinated	57.15	31.38–82.92	<0.01
Apelin (ng/L)	Healthy	99.74	65.28–134.2	<0.01
	Recovered	28.41	0.77–56.05	<0.01
	Mild COVID-19	23.43	14.38–32.48	<0.001
	Severe COVID-19	25.62	18.43–32.82	<0.001
	Vaccinated	32.97	18.27–47.68	<0.01

Legend: Means and IQRs, min/max for ACE2, TMPRSS2, IL-17A, VD, ADAM-17, and AP in the following groups: healthy individuals, immunized individuals, individuals who had recovered, and COVID-19 patients; *p*-values indicate statistical significance.

**Table 3:** Comparison between serum, NPS and saliva.

Biomarker	Group	Means	IQR, min/max	*p*-Value
ACE2 (pg/mL)	Serum	641.3	466–816.7	<0.001
	NPS	508.3	359.1–657.5	<0.01
	Saliva	632.9	279.5–986.3	0.008
TMPRSS2 (ng/mL)	Serum	10.55	9.26–11.84	<0.01
	NPS	7.19	5.43–8.94	0.007
	Saliva	7.99	5.75–10.23	<0.01
IL-17A (ng/L)	Serum	3.8	3.26–4.33	<0.001
	NPS	2.89	1.93–3.86	<0.001
	Saliva	2.26	1.62–2.9	<0.001
Vitamin D (ng/mL)	Serum	187.1	158.3–215.9	<0.001
	NPS	125.6	104.2–147.1	<0.001
	Saliva	152.2	133.6–170.8	<0.001
ADAM-17 (ng/mL)	Serum	60.75	49.15–72.35	<0.001
	NPS	35.81	28.6–43.01	<0.001
	Saliva	35.89	25.82–45.96	<0.001
Apelin (ng/L)	Serum	39	29.31–48.69	<0.001
	NPS	26.21	20.84–31.59	0.06
	Saliva	28.19	22–34.37	<0.001

Legend: Means and IQR, min/max for ACE2, TMPRSS2, IL-17A, VD, ADAM-17, and AP in the following groups: serum, NPS and saliva; *p* values indicate statistical significance.

## Data Availability

Data are contained within the article and Supplementary Materials.

## Supplementary Materials

The following supporting information can be downloaded at: https://www.mdpi.com/article/10.3390/life15020324/s1, Table S1: Characteristics of COVID-19 patients.

## Abbreviations

ACE2, angiotensin-converting enzyme 2; ADAM17, metalloprotease 17; RAAS, renin–angiotensin–aldosterone system; Ang II, angiotensin II; AP, apelin; COVID-19, coronavirus disease 2019; CD4+ T cells, helper T cells; ELISA, enzyme-linked immuno-sorbent assay; IL-17A, interleukin-17A; NPS, nasopharyngeal swab; TMPRSS2, transmembrane serine protease 2; VD, vitamin D.
