# Supplementary material for: Analysis of Biomarker Levels in Nasopharyngeal Swabs, Serum, and Saliva Across Different Health Conditions
Source: Life (Basel). 2025 Feb 19;15(2):324. doi: 10.3390/life15020324 (PMC11857456; doi:10.3390/life15020324)
Supplement: Supplementary file 1 [file life-15-00324-s001.zip › life-3426107-supplementary.pdf]

REPUBLIC OF BULGARIA, Minister of Health, ORDER,
file:///C:/Users/user/Downloads/order_01-977_en_final.pdf

Table S1. Characteristics of individuals

Demographic characteristics									
Mild COVID-19		Cevere COVID-19		Vaccinees		Recovered		Healthy	
N	Median age	N	Median age	N	Median age	N	Median age	N	Median age
102	70 (33-81)	61	78 (68-87)	20	45 (27-60)	19	55 (29-81)	16	54 (35-69)

Data are presented as median and interquartile range (IQR).

Laboratory results of COVID-19 patients					
	Low	High	Mean	95% LCL for Median	95% UCL for Median
Hemoglobin (HGB) %	120	160	133.00	127.00	144.00
Red blood cells (RBCs) %	3.9	5.3	4.56	4.21	4.84
White blood cells (WBCs) %	3.5	10.5	8.08	3.57	9.42
Platelets (PLT) %	140	400	147.00	136.00	283.00
Neutrophil granulocytes (Sg) %	42	70	80.40	81.30	91.00
Monocytes (MONO) %	5.8	11.8	4.65	2.80	4.70
Lymphocytes	1.3	3.9	0.69	0.55	0.70

(LYMPH) %					
Lymphocytes (LYMPH) %	22	48	14.50	6.10	13.30
Westergren (ESR) %	2	30	44.50	24.00	71.00
Fibrinogen (Fbg) %	2.0	4.5	5.45	5.55	7.36
D-Dimer %	0.0	0.50	1.51	0.63	2.27
Glucose (GLUC) %	2.8	6.1	8.40	6.70	7.90
C-reactive protein (CRP) %	0	10	57.00	57.00	88.00
AcAT (AST) %	0	36	50.50	32.00	108.00
ALAT, SGPT %	0	35	27.00	27.00	709.00
Lactate dehydrogenase (LDH) %	36,7	56,7	626.00	133.00	1345.00

Used COVID-19 vaccines.

N	Age	COVID-19 Vaccines	Vaccine Type/Platform	Developer of the Vaccines	Dose
13	38.0 (27.0–40.0)	Pfizer-BNT162b2 Pfizer-BioNTech	Nucleoside-modified mRNA	BioNTech SE, Pfizer Inc.	Two doses given three weeks apart
7	54.0 (47.0–60.0)	Moderna vaccine (mRNA-1273)	Nucleoside-modified mRNA	United States National Institute of Allergy and Infectious Diseases,	Two doses given four weeks apart

				Moderna Inc.	
